# Morbi-mortalité de la varicelle en milieu hospitalier: à propos de 19 cas colligés au Centre Hospitalier Universitaire Yalgado Ouédraogo, Burkina Faso

**DOI:** 10.11604/pamj.2019.33.19.17913

**Published:** 2019-05-13

**Authors:** Jean Claude Romaric Pingdwindé Ouédraogo, Serge Ouoba, Mamoudou Savadogo, Moussa Sawadogo, Edmond Nikiema, Hamadé Ouédraogo, Emmanuel Sawadogo, Panba Sama, Issouf Yaméogo, Flora Coulibaly, Dieu-Donné Ouédraogo

**Affiliations:** 1 Institut de Recherche en Sciences de la Santé (IRSS), Ouagadougou, Burkina Faso; 2 Unité de Recherche Clinique de Nanoro (URCN), Nanoro, Burkina Faso; 3 Université Ouaga1 Prof. Joseph Ki-Zerbo, Ouagadougou, Burkina Faso; 4 Service des Maladies Infectieuses et Tropicales, Centre Hospitalier Universitaire Yalgado Ouagadougou (CHU-YO), Ouagadougou, Burkina Faso; 5 Action Contre la Faim, Diapaga, Burkina Faso; 6 Centre Hospitalier Régional de l'Amitié, Koudougou, Burkina Faso; 7 Service de Maladies Infectieuses, Centre Hospitalier Universitaire Sourou Sanou (CHU-SS), Bobo-Dioulasso, Burkina Faso; 8 District Sanitaire de Gourcy, Gourcy, Burkina Faso; 9 Service de Rhumatologie, Centre Hospitalier Universitaire de Bogodogo (CHU-B), Ouagadougou, Burkina Faso

**Keywords:** Varicelle, hôpital, épidémiologie, enfant, adulte, Varicella (chickenpox), hospital, epidemiology, child, adult

## Abstract

La varicelle est une virose dont l'épidémiologie est mal connue au Burkina Faso. Cette étude s'est donnée pour objectif de décrire le profil hospitalier de la pathologie dans le service des maladies infectieuses du Centre Hospitalier Universitaire Yalgado Ouedraogo (CHU-YO). Il s'est agi d'une série de 19 patients hospitalisés entre le 1^er^ janvier 2005 et le 31 décembre 2014. Ont été inclus, tous les patients avec un dossier complet et exploitable, dont le diagnostic positif de varicelle a été fait. La proportion périodique de varicelle était de 6,2% et a représenté 14,6% des éruptions fébriles. Elle était plus fréquente en 2011, de janvier à mars. L'âge médian était de 19 ans, et la moitié des patients avaient entre 6 et 30 ans. Les comorbidités étaient dominées par l'infection à VIH et l'herpès. Sur le plan clinique, la fièvre et le prurit étaient les symptômes communs et la vésicule la principale éruption. A l'admission, les principales complications étaient pulmonaires, hématologiques et cutanées. La durée médiane d'hospitalisation était de 5 jours, avec des extrêmes de 0 et 13 jours. Les principaux traitements étaient antiviral dans 9 cas, antipyrétique dans 19 cas, local dans 17 cas et antihistaminique dans 11 cas. Sur 19 cas de varicelle, 14 ont guéri et deux sont décédés, dont un adulte sur 10 et 1 enfant sur 9. Si la varicelle est une affection habituellement bénigne, elle peut être fatale chez l'adulte et l'enfant en cas de complications graves.

## Abstract (en)

Varicella is a viral disease whose cause is poorly known in Burkina Faso. The aim of this study is to describe varicella features in the department of infectious diseases at the Yalgado Ouedraogo University Hospital. We here report a series of 19 cases of patients hospitalized between the 1^st^January 2005 and 31^st^ December 2014. The study included all patients with complete and workable medical records who received a positive diagnosis of varicella. The rate of varicella was 6.2%, accounting for 14.6% of febrile rash. It had been more common in 2011, from January to March. The average age was 19 years and half of the patients were aged between 6 and 30 years. Comorbidities were dominated by HIV infection and herpes. Clinically, fever and pruritus were the typical symptoms and vesicles were the most common type of eruption. On admission, the main complications included pulmonary, hematological and cutaneous complications. The median duration of hospitalization was 5 days, ranging between 0 and 13 days. The main treatments were based on antiviral drugs in 9 cases, antipyretic drugs in 19 cases, topic drugs in 17 cases and antihistamines in 11 cases. Out of 19 cases of varicella, 14 were cured and 2 died, including 1 adult out of 10 and 1 child out of 9. Varicella is usually a benign disease. It can be fatal in adults and children with severe complications.

## Introduction

Selon l'Organisation Mondiale de la Santé (OMS), la varicelle est la plus contagieuse des pathologies éruptives et évolue sous un mode endémo-épidémique, avec des épidémies tous les 2 ou 3 ans [1]. Sa transmission est aérienne par le virus varicelle zona, un virus à ADN de la famille des herpesviridae [2]. La varicelle constitue la primo-infection, le zona sa récurrence localisée [2]. La varicelle est répandue à travers le monde; au Burkina Faso, c'est un motif de consultation externe et d'hospitalisation à tout âge dans les centres médicaux et les hôpitaux [1,3]. Elle est plus fréquente chez l'enfant dans les zones tempérées et chez l'adulte en zone tropicale [1,2]. Le diagnostic est avant tout clinique, basé sur la lésion élémentaire qui est la vésicule, et des lésions d'âges différents [2]. La varicelle est une affection bénigne chez l'enfant, grave chez l'adulte, l'immunodéprimé et la femme enceinte, où elle est pourvoyeuse de nombreuses complications potentiellement mortelles [4,5]. Sa prise en charge est avant tout symptomatique [6]. Dans certains pays développés, la vaccination existe et a permis de réduire son incidence et les hospitalisations, ce qui n'est pas encore le cas au Burkina Faso [4,7]. Par ailleurs, bien connue dans les pays développés, l'épidémiologie de la varicelle est encore peu décrite dans les pays en développement, comme le Burkina Faso [1]. Cette étude a pour objectif de décrire le profil épidémiologique, clinique et thérapeutique des patients souffrant de varicelle dans un centre de santé de référence.

## Méthodes

**Cadre de l'étude**: le service des maladies infectieuses du Centre Hospitalier Universitaire Yalgado Ouedraogo (CHU-YO) nous a servi de cadre d'étude. Le CHU-YO est un des centres de référence du pays.

**Type d'étude**: il s'est agi d'une série de 19 patients hospitalisés entre le 1^er^ janvier 2005 et le 31 décembre 2014 dans le service. Tous les cas de varicelle admis dans la période ont été recensés, à partir des dossiers des patients. Ont été inclus, tous les patients ayant un dossier médical complet, chez qui le diagnostic positif de varicelle a été fait. Ce diagnostic est avant tout clinique, basé sur l'anamnèse (chronologie d'apparition des lésions, contage), la fièvre ou une histoire de fièvre et la présence de lésions vésiculaires d'âges différents [2].

**Collecte des données**: une fiche de collecte a été établie pour collecter les données à partir des dossiers médicaux retenus. Les variables suivantes ont été étudiées: épidémiologiques (année d'admission, mois d'admission, âge, sexe, terrain), cliniques (constantes, motifs de consultation, signes physiques à l'admission), paracliniques (numération formule sanguine, transaminases, imagerie), thérapeutiques (voie d'administration, traitement reçu) et évolutives.

**Analyse des données**: les données ont été analysées à l'aide du logiciel IBM SPSS dans sa version 20; les tableaux et les graphiques ont été réalisés à l'aide de Microsoft Office 2016.

## Résultats

**Figure 1:**
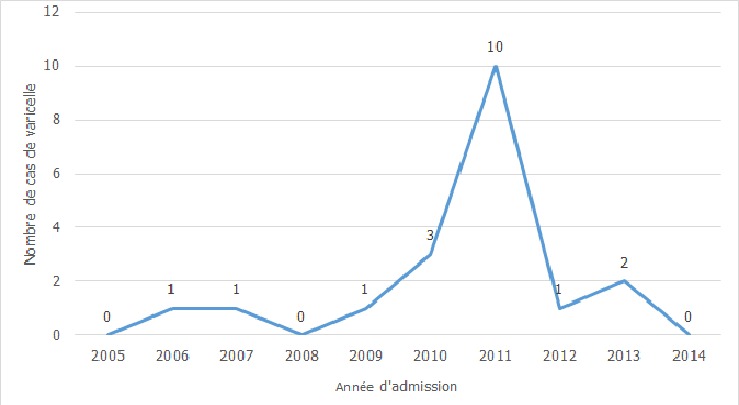
distribution des cas de varicelle selon l'année d'admission, de 2005 à 2014, CHU-YO, Ouagadougou

**Figure 2:**
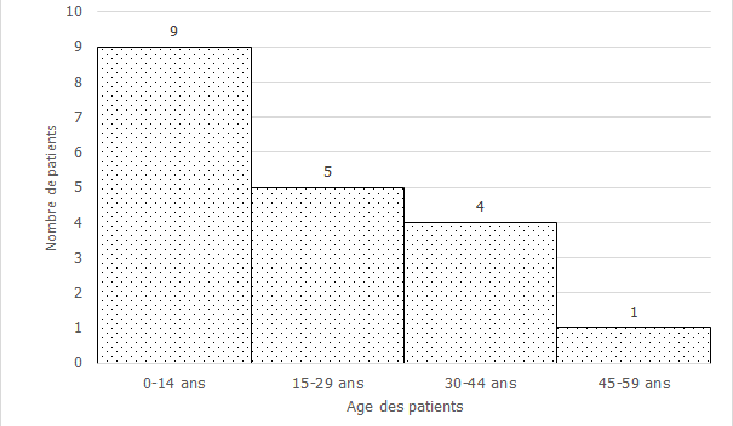
distribution selon l'âge des patients atteints de varicelle, de 2005 à 2014, CHU-YO, Ouagadougou

**Aspects épidémiologiques**: durant la période d'étude, 3084 patients ont été hospitalisés dans le service, dont 19 cas de varicelle, soit une proportion périodique de 6,2‰. Les enfants représentaient 10 cas et les adultes 9. La varicelle a représenté 14,6% des éruptions fébriles. En 2011, il y'a eu 10 cas (Figure 1). Les mois de janvier à mars enregistraient au total 10 cas, avec un (1) ou deux (2) cas par mois pour le reste de l'année. L'âge médian était de 19 ans (q1=6 ans, q3=30 ans). Les première et troisième quartiles étaient respectivement de 6 et 30 ans. Ainsi, 50% des patients avaient entre 6 et 30 ans; en particulier, 25% avaient plus de 30 ans. Les patients dont l'âge était inférieur à 15 ans et ceux dont l'âge était compris entre 16 et 30 ans représentaient 9 et 5 cas respectivement (Figure 2). Le sex-ratio était de 1,1.

**Aspects cliniques et paracliniques**: la co-morbidité était dominée par 3 cas d'infection au virus d'immuno-déficience humaine (VIH) et deux cas d'herpès. Les plaintes étaient dominées par la fièvre et/ou le prurit. Une asthénie profonde a été notée dans 8 cas. La vésicule était présente chez tous les patients, seule ou associée à d'autres lésions élémentaires (papules, bulles, pustules, ulcération etc). Une atteinte muqueuse oropharyngée ou conjonctivale était présente dans 3 cas. Lors de l'admission, des complications pulmonaires (42,1%), hématologiques (42,1%) et cutanées (36,8%), parfois confirmées par des examens biologiques et d'imagerie, ont pu indiquer une hospitalisation. La durée médiane d'hospitalisation était de 5 jours, avec des extrêmes de 0 et 13 jours. Les aspects cliniques et paracliniques sont consignés dans le Tableau 1.

**Tableau 1:** description des paramètres cliniques à l’admission de 19 patients atteints de varicelle, CHU-YO, Ouagadougou

Signes	Effectif (n)	Pourcentage (%)
**Co-morbidités**		
VIH	3	15,8
Herpès	2	10,5
Autres*	6	31,6
**Plaintes principales**		
Fièvre	12	63,1
Prurit	7	36,8
Toux	4	21,1
**Etat général**		
Bon	4	21,1
Assez bon	7	36,8
Altéré	8	42,1
**Conscience**		
Bonne	18	94,7
Obnubilation	1	5,3
Coma	0	0,0
**Eruption cutanée**		
Vésicule	19	100,0
Papule	1	5,3
Bulle	1	5,3
Ulcération	1	5,3
Atteinte muqueuse	3	15,8
**Complications**		
Pulmonaires	8	42,1
Hématologiques	8	42,1
Cutanées	7	36,8
Rénales	5	26,3
Hépatiques	2	10,5
Neurologiques	1	5,3

* Autres: Abcès cérébral, insuffisance tricuspidienne, asthme, syndrome néphrotique, HTA, grossesse

**Aspects thérapeutiques et évolutifs**: les aspects thérapeutiques sont consignés dans le Tableau 2. L'évolution s'est faite majoritairement (14/19cas) vers la guérison, dont 8/9 enfants et 6/10 adultes. Elle s'est faite sans rémission clinique notable chez 3 adultes sur 10. Deux décès ont été rapportés, soit 1/9 chez les enfants et 1/10; ils ont concerné des patients atteints au moins de complications simultanées pulmonaires et cutanées à l'admission. Chez les patients guéris, aucune séquelle n'a été décrite à la sortie.

**Tableau 2:** distribution selon les aspects thérapeutiques de 19 patients atteints de varicelle, CHU-YO, Ouagadougou

Aspects thérapeutiques	Effectif(n)	Pourcentage (%)
**Voies d’administration**		
Entérale	7	36,8
Parentérale et entérale	7	36,8
Parentérale	5	26,3
Locale	17	100,0
**Traitement antiherpétique**		
Acyclovir	9	47,4
**Traitement antipyrétique**		
Paracétamol	19	100,0
**Traitement dermatologique**		
Traitement antiseptique local	17	100,0
**Traitement antihistaminique**		
Antihistaminique H1	11	52,6
**Traitements adjuvants**		
Traitement mucolytique	2	10,5
Traitement antibiotique	2	10,5
Traitement anti-anémique	2	10,5
Traitement antirétroviral	1	5,3
Traitement antihypertenseur	1	5,3
Corticothérapie	1	5,3

## Discussion

Toute interprétation de nos résultats doit tenir compte des biais liés à la faible taille de notre échantillon et du recrutement hospitalier expliquant la fréquence des formes compliquées dans notre série.

**Aspects épidémiologiques**: la proportion hospitalière de la varicelle de 2005 à 2014 est de 6,2‰ au CHU-YO, habituellement plus élevée que dans les séries européennes, même à l'ère prévaccinale, pouvant atteindre 7,6/100000 personnes-années ou 0,6-1,5/100000 personnes [4,8]. La varicelle est une pathologie généralement bénigne, si bien que certaines hospitalisations sont dues aux complications [7-9]. Ainsi, la forte proportion observée dans notre série pourrait être liée à la nature hospitalière de nos données, du fait que seules les formes compliquées de la maladie ont été admises en hospitalisation. Si la vaccination contre la varicelle est implémentée dans certains pays européens ou américains, et a permis de réduire l'incidence et les complications dues à la varicelle, elle n'est pas encore inscrite dans le programme élargi de vaccination (PEV) au Burkina Faso [1,4,10]. En fait, même si l'OMS a recommandé le vaccin contre le virus varicello-zonateux dans les vaccins de routine chez l'enfant depuis 1998, il est encore rarement utilisé en Afrique [11,12]. L'année 2011 constitue-t-elle une année épidémique? Une série chronologique pourrait y répondre. Les cas de varicelle étaient plus fréquents entre janvier et mars, des mois secs et froids, plus propices à sa transmission qui se fait par voie aérienne et par contact direct [1]. En effet, ce temps favorise l'assèchement des muqueuses nasales à un moment où les rhinites virales seraient fréquentes, avec des risques de microlésions. Celles-ci constituent de potentielles portes d'entrées pour le virus de la varicelle et du zona qui pénètre l'organisme par les voies respiratoires supérieures, avant d'évoluer par voie hématogène [2]. La varicelle est potentiellement grave chez le nourrisson, la femme enceinte et l'adulte, avec un risque accru d'hospitalisation [1,2]. Ce qui expliquerait qu'au moins 50% des patients de notre série hospitalière soient adultes; l'âge médian était de 19 ans. Par ailleurs, la maladie surviendrait souvent à un âge plus tardif en zones tropicales, au contraire des zones tempérées où la grande majorité des adolescents et des enfants font la séroconversion avant l'âge adulte [1,2,5,13]. Toutefois, les raisons de ces différences sont mal élucidées et seraient liées au climat, à la densité de la population et au risque d'exposition [1]. La survenue à un âge tardif augmenterait des risques de formes graves et de mortalité associée.

**Aspects cliniques et paracliniques**: certaines co-morbidités telles que l'infection à VIH, l'insuffisance tricuspidienne, l'hypertension artérielle, le syndrome néphrotique, l'asthme et la grossesse favorisent des formes graves ou compliquées de la varicelle, augmentant le risque d'hospitalisation, voire de complications [1,2,14]. La varicelle est une maladie éruptive dont la lésion élémentaire est la vésicule qui évolue par poussées successives pour aboutir à des lésions d'âges différents, souvent associées à des lésions muqueuses [2]. Elle associe une fébricule habituellement et un prurit intense qui, à travers les lésions de grattage favoriserait la surinfection cutanée, surtout chez les enfants [2,4]. La toux, les lésions bulleuses et l'altération de la conscience sont des signes de complications pulmonaires, cutanées, ou de gravité [2,4,6]. A l'admission, les complications les plus fréquentes étaient pulmonaires, hématologiques et cutanées. Les atteintes neurologiques et pulmonaires sont plus fréquentes chez l'adulte, les atteintes cutanées chez l'enfant, les autres étant rares [4,5]. D'après Pierik *et al.* les complications pulmonaires peuvent être fréquentes en consultation [15]. En particulier, elles sont un facteur pronostic important et justifient la mise en route d'un traitement antiviral précoce [4,6].

**Aspects thérapeutiques et évolutifs**: le traitement de la varicelle se décline en traitements antiviral et symptomatique. La voie entérale a prédominé, seule ou en association avec la parentérale pour répondre aux conditions cliniques des patients. Des particularités cliniques telles que la néo-natalité, la grossesse, les pneumopathies ont pu indiquer l'emploi de l'acyclovir, la voie intraveineuse étant réservée aux cas graves [4,6]. Initié précocement, le traitement antiviral réduit les risques de complications [6]. Il faut garder à l'esprit qu'après administration per os, seulement 20% d'acyclovir sont résorbés, contrairement à la voie intraveineuse [16]. Cependant, avec celle-ci les concentrations élevées peuvent être néphrotoxiques, nécessitant une administration lente, en au moins 1 heure [16]. Au traitement antiviral s'associe des traitements antipyrétique, local et antihistaminique. Ainsi, le paracétamol était utilisé chez tous les patients, conformément aux recommandations [6]. En effet, l'emploi de l'aspirine dans la varicelle constitue un risque important de syndrome de Reye et les anti-inflammatoires non stéroïdiens favorisent la survenue de fasciite nécrosante et de surinfections bactériennes [1,9,17]. Le traitement local, à travers des antiseptiques liquides (chlorhexidine, éosine, hexamidine), ainsi que le traitement antihistaminique (chlorpheniramine, cétirine) sont d'autres moyens de prévention des surinfections cutanées, surtout chez les enfants [6]. La létalité due à la varicelle est habituellement faible [1]. Cependant, les complications peuvent grever le pronostic, comme dans notre série où les décès étaient des cas compliqués sur les plans pulmonaire et cutané. Chez l'enfant, la présence de terrain pathologique particulier ou de varicelle grave augmente la mortalité [1, 7]. La mortalité infantile était faible dans notre série, comparée à celle trouvée par Wateba *et al.* sur des cas graves uniquement, mais élevée devant les séries européennes [7,14]. La durée médiane d'hospitalisation était courte (5 jours), mais elle a pu être élevée chez certains, en raison notamment de la gravité des cas.

## Conclusion

La varicelle demeure une maladie bénigne de l'enfant, conduisant rarement à l'hospitalisation. Toutefois, des complications peuvent survenir en cas de co-morbidité ou chez les adultes. Celles-ci pourraient aggraver le pronostic aussi bien chez l'enfant que l'adulte, même en milieu sanitaire de référence. Si la petite taille de notre série et son caractère hospitalier limitent la validité externe de nos résultats, cette étude constitue une source d'hypothèses pour des études d'incidence et de prévalence de la varicelle au Burkina Faso, où son fardeau reste peu connu.

### Etat des connaissances actuelles sur le sujet

- La varicelle est une affection habituellement bénigne;
- La létalité due à la varicelle est faible;
- La co-morbidité augmente le risque de complications.

### Contribution de notre étude à la connaissance

- La proportion d'adultes hospitalisés pour varicelle est élevée;
- L'association de complications pulmonaires et cutanées peut être source de létalité, quel que soit l'âge;
- La co-morbidité est dominée par l'infection à VIH au Burkina Faso.

## Conflits des intérêts

Les auteurs ne déclarent aucun conflit d'intérêts.
